# Draft Genome Sequence of *Microcystis aeruginosa* NIES-98, a Non-Microcystin-Producing Cyanobacterium from Lake Kasumigaura, Japan

**DOI:** 10.1128/genomeA.01187-16

**Published:** 2016-11-10

**Authors:** Haruyo Yamaguchi, Shigekatsu Suzuki, Tomoharu Sano, Yuuhiko Tanabe, Nobuyoshi Nakajima, Masanobu Kawachi

**Affiliations:** a Center for Environmental Biology and Ecosystem Studies, National Institute for Environmental Studies, Ibaraki, Japan; b Center for Environmental Measurement and Analysis, National Institute for Environmental Studies, Ibaraki, Japan; c Faculty of Life and Environmental Sciences, University of Tsukuba, Ibaraki, Japan

## Abstract

*Microcystis aeruginosa* is a well-known bloom-forming cyanobacterium. We newly sequenced the whole genome of *M. aeruginosa* NIES-98, which is a non-microcystin-producing strain isolated from Lake Kasumigaura, Japan. The genome contains approximately 5.0 Mbp, with an average G+C content of 42.41% and 5,140 predicted protein-coding genes.

## GENOME ANNOUNCEMENT

*Microcystis aeruginosa* is a well-known bloom-forming cyanobacterium in freshwater lakes worldwide (1). The species is genetically divided into at least eight clades (groups A to G and X) based on a multilocus phylogenetic analysis (2). Group A, X, and some group B strains produce hepatotoxic cyanotoxins called microcystins. *M. aeruginosa* NIES-98 is a non-microcystin-producing strain isolated from Lake Kasumigaura, Japan (2). The strain is available via the Microbial Culture Collection at the National Institute for Environmental Studies in Japan (http://mcc.nies.go.jp/). According to the previous phylogenetic analysis by Tanabe and Watanabe (2), NIES-98 is a group B strain. Thus far, whole-genome sequencing of only three strains of *M. aeruginosa* from Lake Kasumigaura have been reported: NIES-843 (group A) (3), NIES-44 (group E) (4), and NIES-2549 (group G) (5). Additional genetic information on *M. aeruginosa* is required to determine the spatial and seasonal dynamics in Lake Kasumigaura and the evolutionary history of this species.

Our analysis of volatile compounds using gas chromatography-mass spectrometry (GC-MS) showed that NIES-98 produces 3,5-dimethylanisole, which is known as a kind of fungal volatile compound (6); however, the other major volatile compounds from this strain remain unidentified.

Genomic DNA was extracted from 50 ml of the axenic NIES-98 culture using a DNeasy plant minikit (Qiagen) and fragmented to approximately 550-bp segments using a Covaris M220 ultrasonicator (Covaris). The genomic library was constructed using a TruSeq Nano DNA library prep kit for NeoPrep (Illumina) and sequenced by the MiSeq platform (Illumina) using the 600-cycle MiSeq reagent kit version 3. The number of resultant paired-end reads was 5,681,573. Low-quality reads/bases were filtered using Trimmomatic version 0.36 (7), and *de novo* assembly was performed using SPAdes 3.9.0, with k-mer of 21, 33, 55, 77, 99, and 127 (8). The resulting draft genome comprises 497 scaffolds (>200 bp in size) of 4,983,728 bp, with an average genome coverage of approximately 322.1×. The maximum scaffold length was 199,403 bp, and the mean size of the scaffolds was 10,027 bp. The draft genome of NIES-98 was annotated using the RAST server (9). The genome comprises 5,140 predicted protein-coding sequences (CDSs) including 2,236 hypothetical proteins, and 50 RNA genes. The number of CDSs of NIES-98 is larger than those of NIES-44 (4,790 CDSs) and NIES-2549 (4,282 CDSs) but smaller than that of NIES-843 (6,312 CDSs). The G+C content of the genome is 42.41%. Functional annotation based on COG categories using COGNIZER (10) showed that NIES-98 has a smaller number of genes for transposase and inactivated derivatives, including those in category L, than the three aforementioned strains. A microcystin biosynthesis gene cluster was not detected in the genome of NIES-98, as shown in a previous study (11). antiSMASH (12) predicted 11 secondary metabolite gene clusters in the genome, including aeruginosin, puwainaphycins, and micropeptin biosynthetic gene clusters. This genomic sequence provides basic information for better understanding of the ecology and evolution of *M. aeruginosa*.

### Accession number(s).

This whole-genome shotgun project has been deposited in GenBank under the accession no. MDZH00000000. The version described in this paper is the first version.
